# Optimizing Gold Nanoparticle Size and Shape for the Fabrication of SERS Substrates by Means of the Langmuir–Blodgett Technique

**DOI:** 10.3390/nano10112264

**Published:** 2020-11-16

**Authors:** Mohammad Tahghighi, Davide Janner, Jordi Ignés-Mullol

**Affiliations:** 1 Departament de Química Fíisica, Universtitat de Barcelona, 08028 Barcelona, Spain; mohamadtahghighi06@yahoo.com; 2 Institute of Nanoscience and Nanotechnology, Universtitat de Barcelona, 08028 Barcelona, Spain; 3 Dipartimento di Scienza Applicata e Tecnologia (DISAT) and RU INSTM, Politecnico di Torino, 10129 Torino, Italy; davide.janner@polito.it

**Keywords:** SERS, Langmuir–Blodgett, non-spherical nanoparticles

## Abstract

The Langmuir–Blodgett technique, in which a layer of nanoparticles is spread at the water/air interface and further transferred onto a solid support, is a versatile approach for the preparation of SERS substrates with a controllable arrangement of hotspots. In a previous work, we demonstrated that fine-tuning the lateral packing and subsequent seed growth of 10 nm gold nanoparticles led to a quasi-resonant enhanced in the SERS signal of a test analyte. Here, we explore further enhancements by modifying the size and shape of the spread gold nanoparticles in order to take advantage of the inherent interparticle repulsion mechanisms present at the interface. We show that the size of the used nanoparticles is also a determinant factor, which cannot be compensated by the subsequent electroless growth. We also show that, although the seeded growth leads to rough hotspots, the sensitivity can be optimized by self-assembling urchin-shaped nanoparticles, with a roughness that is fine-tuned a priori. Our results suggest an intriguing correlation between surface homogeneity and SERS signal enhancement, indicating that regular substrates will have the optimal performance.

## 1. Introduction

Surface Enhanced Raman Spectroscopy (SERS), a leading label-free molecular-detection technique, is extremely sensitive to surface nanostructuring, and many current efforts are focused at developing SERS substrates that contain a lattice of hotspots, i.e., localized nanostructures where the light is trapped by the local plasmonic field, which allows single-molecule detection. Among the leading factors that influence the SERS performance of these substrates, the material, size, shape, and packing density are directly influenced by the manufacturing technique [1,2].

The use of bottom-up wet chemistry methods for the development of functional nanostructured materials is a scalable and cost-efficient approach that relies on the self-assembly of pre-synthesized components [3,4,5]. Typically, nanoparticles (NPs) are the chosen building blocks, whose size, shape, and composition are selected based on the target physical properties of the final device [6]. These strategies have also been often employed for the development of SERS substrates [7,8,9,10,11,12,13,14,15,16,17,18].

Among the self-assembly manufacturing methods, the Langmuir–Blodgett (LB) technique stands out for its versatility and tunability of the lateral packing of nanoscale constituents. This technique was originally developed for the coating of glass substrates with a monolayer of insoluble surfactants previously organized at the water/air interface, where their packing density can be easily adjusted by means of moving barriers [19]. The film can be finally deposited on to target surface by sliding the latter through the interface, with the sole requirement that the substrate is able to drag a thin layer of the aqueous subphase. This original technique has been amply used in recent years for the manufacturing of nanomaterials as well, since nanoparticles with suitable surface chemistry, rather than molecular entities, can be spread at the water/air interface, tuned for lateral packing, and further transferred onto a substrate. The LB method has also been used to prepare SERS substrates, either directly [7,9,10,11] or with further processing [8,18], where the transferred nanoparticles act as seeds for the growth of hotspots [12]. A complementary transfer technique, the Langmuir–Shaefer method, has also been demonstrated for the preparation of SERS substrates. In this case, the particles are similarly spread and arranged at the water/air interface, but the transfer is achieved on a substrate that is put into contact from the air side of the interface, without penetrating the aqueous subphase. This alternative transfer method relieves the requirement that the substrate be hydrophilic, thus allowing to design a tighter chemical compatibility with the nanoparticles.

In a recent work [12], we demonstrated that the Langmuir–Blodgett technique is a simple and versatile approach to build SERS substrates through the tailored self-assembly of commercial spherical gold nanoparticles. In that work, we found that the signal gain produced by the plasmonic substrate strongly depended upon the lateral density and the size of the hotspots, leading to a quasi-resonance for a narrow set of experimental conditions. Our original approach employed commercial 10 nm spherical gold NPs that were deposited as a LB film and subsequently used as seeds for an electroless deposition of gold, which resulted in nonspherical hotspots with a larger effective final size. In view of that result, we explore here the effect of employing gold NPs of different sizes to prepare gold NP monolayers and find the optimal core size to manufacture SERS substrates. Since, after gold deposition, the hotspots in the monolayers were rough, we also address here the possibility to enhance the substrates by employing custom-made non-spherical NP where the hotspot roughness may be controlled. We find that, for spherical NPs, those with intermediate size lead to substrates with the optimal balance of packing density and roughness for SERS detection of a test analyte. Moreover, we demonstrate that the SERS performance can be significantly improved by using urchin-shaped, instead of spherical, gold NPs to prepare the LB films. Finally, we demonstrate the feasibility of using our optimal substrates in the real case of detecting a water pollutant.

## 2. Experimental Section

### 2.1. Materials

Sperical gold NPs were purchased as aqueous suspensions from Sigma-Aldrich. 1-dodecanthiol (DDT, ≥98%), poly(ethyleneglycol)methyl ether thiol (PEG-SH, 6000 g/mol), 4-mercaptobenzoic acid (4-MBA, 99%), Bis(dimethylthiocarbamoyl) disulfide (Thiram—Analytical Standard), hexane (98.5%), hydrogen peroxide (30%), (3-aminopropyl) triethoxysilane (APTES, 99%), Gold(III) chloride trihydrate (HAuCl${}_{4}$·3H${}_{2}$O, 99.99%), sodium citrate tribasic dihydrate (≥98%), hexadecyltrimethylammonium bromide (CTAB, ≥99%), silver nitrate (AgNO${}_{3}$, ≥99%), and L-ascorbic acid (AA, ≥99%) were purchased from Sigma Aldrich (Darmstadt, Germany). Ethanol 96%, sulfuric acid (95%), and hydrochloric acid (37% *v/v*) were purchased from Panreac (Barcelona, Spain). Reagent grade toluene was purchased from Scharlau (Barcelona, Spain). All chemicals were used as received. Polished 7980 fused-silica slides (2SPI, West Chester, PA, USA) were used as substrates for LB transfer and subsequent measurements. Milli-Q water was used in all suitable processes.

### 2.2. Methods

#### 2.2.1. Nanoparticle Preparation

While spherical gold NPs were used as received in their aqueous suspensions, urchin-shaped gold NPs were in-house synthesized following the protocols described by Serrano et al. [10]. In brief, we mixed 500 μm of 20 μm gold NP suspension, 10 mL of 0.25 mM HAuCl${}_{4}$, and 10 μL of 1.0 M HCl in a glass vial, under moderate stirring. We quickly added 100 μL of AgNO${}_{3}$ (3 mM) and 50 μL of ascorbic acid (100 mM) to the solution and the color of the mixture changed from pink to blue and purple, indicating a change in the particles size and shape. The final size of the urchin-shaped nanoparticles was verified with TEM.

In order to spread the NPs at the water/air interface, they were first transferred from the original aqueous solution to a chloroform suspension using a the seed-mediated growth protocol described by Serrano et al. [10]. In brief, PEG-SH was added to the aqueous particle dispersion at a nominal concentration of 0.8 PEG-SH molecules per nm${}^{2}$ of particle surface, which we estimated from the nominal particle size and concentration provided by the manufacturer. The aqueous solution, to which 3 μL per mL of concentrated HCl was added, was put inside a test tube along with the same volume of a DDT chloroform solution with a nominal concentration of 148 DDT molecules per nm${}^{2}$ of particle surface. A total of 20 min of vigourous stirring led to the particle exchange across the water/chloroform interface, yielding the chloroform particle suspension required for Langmuir monolayer preparation. Excess amount of thiol compounds were removed by three cycles of washing and centrifuging (4500 rpm, 30 min) with fresh Milli-Q water. All these procedures were carried out at room temperature. With this protocol, the final concentration of NP in chloroform was the same as in the original aqueous suspension.

#### 2.2.2. Langmuir–Blodgettry and Substrate Preparation

Fused-silica slides were modified by silanization with APTES to promote adhesion of gold NPs. After cleaning and oxidizing the substrates with piranha solution (7:3 *v/v* solution of H${}_{2}$SO${}_{4}$ and H${}_{2}$O${}_{2}$), ethanol, and acetone to remove organic contamination, the plates were rinsed with Milli-Q water and finally dried under a nitrogen flow. Clean and dry plates were immersed in a solution of APTES in toluene 10% *v/v* at 60 ${}^{\circ }$C for 120 min. The functionalized plates were rinsed with toluene, methanol, and finally with Milli-Q water, and dried under a stream of nitrogen. Previously used slides could be reused several times by cleaning them with aqua regia to remove the deposited gold layer.

Gold NP monolayers were spread by dropwise deposition of the chloroform NP solution onto the water surface in a Langmuir film balance (KSV mini-trough system) at room temperature [19]. After solvent evaporation, functionalized gold particles remain at the water/air interface. A 1×2 cm${}^{2}$ rectangular filter paper plate was hanged from an electrobalance and placed in contact with the water/air interface, thus allowing to measure the surface tension drop (surface pressure) in real time (Wilhelmy plate method). Additional monitoring was achieved by means of a custom made Brewster angle microscope (BAM). The reflectivity from the interface scales with the square of the thickness of any adsorbed layer, thus allowing to assess the formation of multilayer domains and to ensure that the spread NP have reached a stationary distribution prior to compression. Monolayers were transferred at a constant lateral pressure, which is achieved by closing the barriers of the Langmuir trough at 0.1 mm s${}^{-1}$, using the Langmuir–Blodgett technique. In brief, the clean and prepared slide is dipped into the water subphase prior to monolayer spreading, followed by vertical extraction when the desired monolayer state is achieved. Extraction was carried out at a constant speed of 0.1 mm s${}^{-1}$ using the dip-coating instrument of the KSV minitrough, which provides a feedback mechanism and control software to compensate for particle loss during plate extraction.

In order to enhance the Raman signal during subsequent SERS analysis, nanoparticle monolayers were used as seeds for the electroless deposition of gold [20]. In brief, NP-coated plates were soaked in a 10 ml solution of 0.01% (*w/v*) HAuCl${}_{4}$, and mixed with 500 μL of hydrogen peroxide in order to reduce gold chloride ions to atomic gold. This electroless plating process was conducted under vigorous agitation of the mixture at room temperature for 8 min. The resulting gold films were cleaned with O${}_{2}$ plasma to remove traces of organic compounds prior to exposing them to chemicals to be detected by Raman measurements.

#### 2.2.3. Analysis Techniques

Scanning Electron Microscopy observations were performed with a Jeol J-7100F Field Emission Scanning Electron Microscope (Tokio, Japan). Samples were assembled on the holder by means of a double sided conductive adhesive disk. A thin carbon layer was evaporated on the samples to improve their electric conductivity.

Raman spectra were obtained using the Raman Dispersive Spectrometer Jobin-Yvon Labram HR 800 (Kyoto, Japan), coupled to an Olympus BXFM optical microscope (Barcelona, Spain). The used detector was a 16-bit dynamic range Peltier cooled CCD. In all the measurements, we illuminated at a wavelength of 532 nm, with a laser power on the sample in the range 0.5–4 mW, using a diffraction grating of 600 lin mm${}^{-1}$ and 100× or 50× microscope objectives. Measurement times for 4-MBA and Thiram were 5 and 20 s, respectively.

To assess the substrates’ SERS performance, a droplet of the analyte solution (4-MBA or Thiram) was deposited on the SERS substrates and we waited until the solvent was fully evaporated. The substrates were finally gently rinsed with Milli-Q water, and dried under a stream of nitrogen. For 4-MBA SERS measurements, the analyte was 300 μL of a 10 μM 4-MBA aqueous solution, prepared by diluting a 1 mM stock solution in ethanol. SERS spectra were obtained within 1 h of 4-MBA monolayer formation. We tested also the stability of the 4-MBA films on the gold and found no significant variation in the signals 24 h after 4-MBA incubation at room temperature under vacuum.

For Thiram detection, a stock solution of 10 mM of pure product in ethanol was prepared. The stock solution was then diluted with MilliQ water to 1 μM and used to deposit the droplet as described above.

The software Labspec (version 5, Horiba Ltd., Kyoto, Japan) and IgorPro (version 8, Wavemetrics, Inc., Tigard, OR, USA) were used to process the SERS spectra. All spectra have been normalized by the laser power. For 4-MBA detection, a band-pass filter with two windows centered at the two main peaks of this substance have been applied to the raw data. For Thiram detection, a moving average filter (5 points) was applied to the raw data.

## 3. Results and Discussion

### 3.1. Monolayers of Spherical Gold Nanoparticles

**Figure 1:**
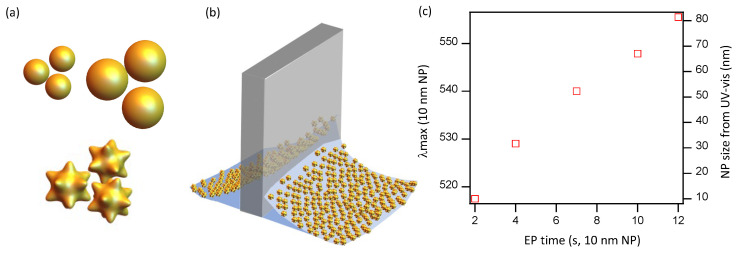
(**a**) Gold nanoparticles (NPs) of different spherical size or star-like shape are spread at the air/water interface and transferred onto a solid plate by means of the Langmuir–Blodgett technique (**b**). (**c**) Maxmimum wavelength of UV–vis absorption spectra of monolayers of 10nm gold NPs that act as seeds, after different gold electroless plating time. The spectrum is compared with that of gold NP of different sizes to provide with a rough estimation of the hotspot size.

In our previous work studying LB films prepared using commercial gold NPs of 10 nm [12], we estimated the size of the hotspots after different amounts of gold electroless plating was applied on the bare NP layer by comparing the UV–vis spectrum of our substrate with that of gold NP of different sizes, as reported by the NP manufacturer (Figure 1). We found that, for the optimal electroless plating time (8–9 min), the average effective hotspot size was around 50 nm. The absorption spectrum of rough NP has a red shift with respect to smooth NP [10], so the spherical core of the optimal hotspot size should, in reality, be significantly lower than 50 nm. In consequence, one should expect that the optimal SERS substrates should be prepared with gold NP of size smaller than 50 nm. In the present study, we used commercial spherical gold NPs of sizes 5–40 nm, and studied the performance of LB films prepared at different lateral pressures for each particle size. For the final SERS substrates, we increased the size and roughness of the hotspots by applying a common gold electroless plating time of 8 min, which was found to be near optimal in our previous study [12].

Functionalized NP are carefully spread at the clean water/air interface (nominal pH = 5.7, in equilibrium with atmospheric CO${}_{2}$) by depositing microdrops of a NP suspension in chloroform (nominal concentration $5\times 10^{16}$ particles/μL) using a Hamilton glass syringe. Drops must be deposited on the interface so that the liquid spreads and evaporates, leaving the dispersed NP trapped in the quasi-two-dimensional environment. The state of the monolayers is monitored by means of the interfacial tension reduction (surface pressure), which is measured in real-time by means of a Wilhelmy film balance. Capillary-mediated interparticle interactions and repulsion between electrical double layers forming in the submerged portion of NP lead to a direct relation between lateral packing and effective surface pressure, so we actively control the latter as a means to obtain NP layers with a reproducible value of the lateral packing density.

NP monolayers at the water/air interface are metastable (as molecular monolayers also often are [19]), and films may collapse into the third dimension above a critical surface pressure. Since SERS is a surface-based phenomena, multilayers do no provide any significant enhancement and are to be avoided as a material-wasting effect. Our experimental setup is equipped with a Brewster angle microscope, which allows to qualitatively monitor the average monolayer thickness and allows to detect the onset of multilayer formation, thus helping to set the upper limit for the usable surface pressures.

**Figure 2:**
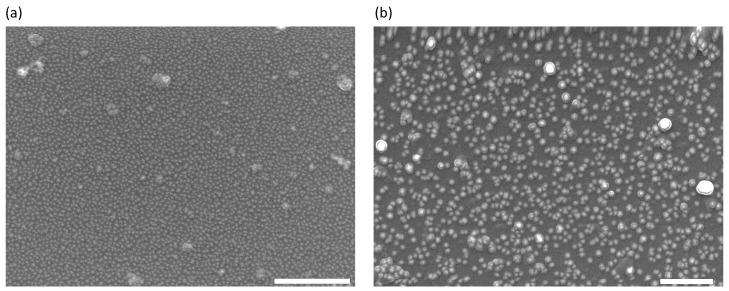
SEM micrographs of spherical gold NP Langmuir–Blodgett (LB) monolayers prepared at 12 mN m${}^{-1}$ of lateral pressure and without further gold electroless plating. Images correspond to NPs of 5 nm (**a**) and 40 nm (**b**). Scale bars are 500 nm.

The quality of the obtained NP monolayers can be assessed by means of SEM imaging (Figure 2). The achievable packing densities decrease with NP size, since stronger capillary effects result in enhanced inter-particle repulsion. It is, however, not obvious that the higher hotspot density in Figure 2a with respect to Figure 2b should result in an enhanced SERS effect, since the size of the hotspots has also an influence on the final plasmonic effect. In these SEM micrographs we observe the absence of NP multilayers, in agreement with BAM monitoring of the water/air interface, and assessing the faithful transfer onto a solid support by means of the LB technique. The few nanoscale clusters that can be observed correspond to aggregates that either existed in the original NP suspension or that formed upon spreading at the water/air interface. Nevertheless, their effect on the final SERS performance should be small.

### 3.2. SERS Performance of Spherical Gold Nanoparticle Monolayers

**Figure 3:**
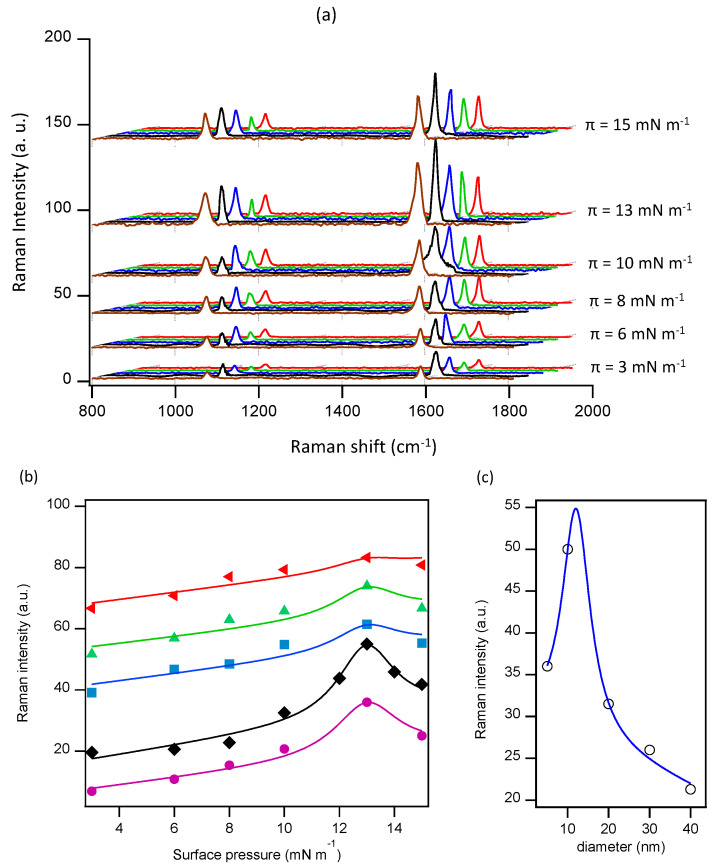
(**a**) Surface Enhanced Raman Spectroscopy (SERS) spectra of 4-MBA adsorbed on gold spherical NP monolayers prepared at different lateral pressures (as indicated in each series) and 8 min of gold electroless plating time. For each series, and from front to back, NP sizes are 5 nm, 10 nm, 20 nm, 30 nm, and 40 nm. (**b**) Height of the SERS peak at 1588 cm${}^{-1}$ for the data in (**a**). From bottom to top, NP size is ordered as in (**a**). (**c**) Height of the SERS peak at 1588 cm${}^{-1}$ and a surface pressure of 13 mNm${}^{-1}$, as a function of NP size. The lines through data are guides for the eye.

To study the influence of NP size and lateral pressure on the SERS performance of the prepared nanostructured surfaces, we have chosen 4-MBA as the standard target analyte. In Figure 3a, we show the Raman spectra for the full set of data, comparing, at each surface pressure, the curves for all studied NP sizes. A common gold electroless plating time of 8 min has been applied to all NP monolayers. We have improved the data by using a band-pass filter that highlights the peaks at 1076 cm${}^{-1}$ and 1588 cm${}^{-1}$, characteristic of 4-MBA. We observe that the optimal signal in all the experiments corresponds to 10 nm NP at a lateral pressure of 13 mN m${}^{-1}$ although, at lower lateral pressures, the height of the peak is higher for 20 nm nanoparticles. Under these experimental conditions, the variation of the lateral pressure has the strongest influence in monolayers of 5 nm and 10 nm NP, while this effect is much more subtle for larger NP (Figure 3b), where the optimal surface pressure leads to less than a 20% improvement in signal strength. By comparing the height of the 1588 cm${}^{-1}$ peak for all NP sizes, we estimate that the optimal signal would be obtained for NP slightly larger than 10 nm (Figure 3c). Nevertheless, the predicted further enhancement would be small with respect to 10 nm NP, and fine tuning the electroless plating time for 10 nm NP may be a better option to seek the optimal performance of spherical gold NPs.

The above result show that our original hypothesis, which assumed a better performance using monolayers of spherical NP whose size was close to the estimated size of the optimal hotspots obtained from the electroless plating of 10 nm NP, was not accurate. While the density of hotspots can be much higher for smaller NP (Figure 2), the final size achievable through electroless plating is limited by the merging of neighboring NP. On the other hand, growth through electroless plating results in significantly rougher entities when compared with similarly sized NP [12], leading to an enhanced SERS effect. We conclude that, tn spite of its flexibility, the Langmuir–Blodgett technique followed by EP growth cannot be improved past the reported results. To increase the performance, in the next section we change the particle geometry, and employ urchin-shaped particles, where the roughness is optimized during NP synthesis, rather than in the EP stage of substrate preparation.

### 3.3. SERS Performance of Urchin-Shaped Gold Nanoparticle Monolayers

**Figure 4:**
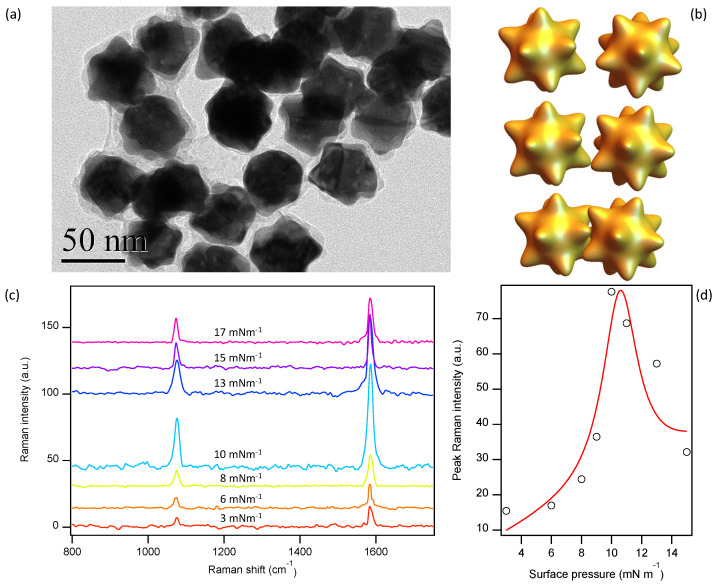
(**a**) TEM image of synthesized gold nanostars. (**b**) Sketch of gold nanostars as the lateral packing density is increased, leading to overlaps that degrade the SERS performance. (**c**) SERS spectra of 4-MBA adsorbed on gold nanostars prepared at different lateral pressures and 8 min of gold electroless plating time. (**d**) Height of the SERS peak at 1588 cm${}^{-1}$ at different surface pressures. The line through data is a guide for the eye.

Using well-established protocols [10], we have synthesized urchin-shaped gold NP, henceforth called gold *nanostars*. In brief, gold is grown on spherical gold NP cores, resulting in the formation of sparse random spikes several nm in height and in width that cover the originally smooth surface. In the experiments shown below, the resulting NP size is about 50 nm in diameter (Figure 4a). The protocol leading to NP functionalization and Langmuir–Blodgett monolayer preparation is identical to the one used for spherical NP. The rough nanostar shapes would enable spike interdigitation between neighboring NP at high packing densities (Figure 4b), which would degrade the SERS performance of the resulting hotspots [10,21]. In consequence, we expect an optimal performance at lower surface pressures compared to our results with monolayers of smooth NP.

Similarly to what we observed using smooth NP, the height of the 4-MBA peaks increases monotonically with surface pressure until a maximum is reached at around 10 mN m${}^{-1}$ (Figure 4c,d) a value sensibly lower than for spherical NP, consistently with our expectations that high packing densities could result in a downgraded performance due to nanostar interdigitation. Remarkably, the height of the 1588 cm${}^{-1}$ peak at optimal performance is significantly higher that the best we could achieve using spherical NP (Figure 3c), evidencing that carefully adjusting the roughness of the hotspots is crucial to achieve optimal performance of the SERS substrates obtained through the Langmuir–Blodgett technique. Although gold electroless plating of the NP monolayer increases the roughness, the growth process is clearly different from the one achieved during nanostar synthesis, and much smoother surfaces are obtained.

### 3.4. Homogeneity of the SERS Substrates and Detection of Thiram

**Figure 5:**
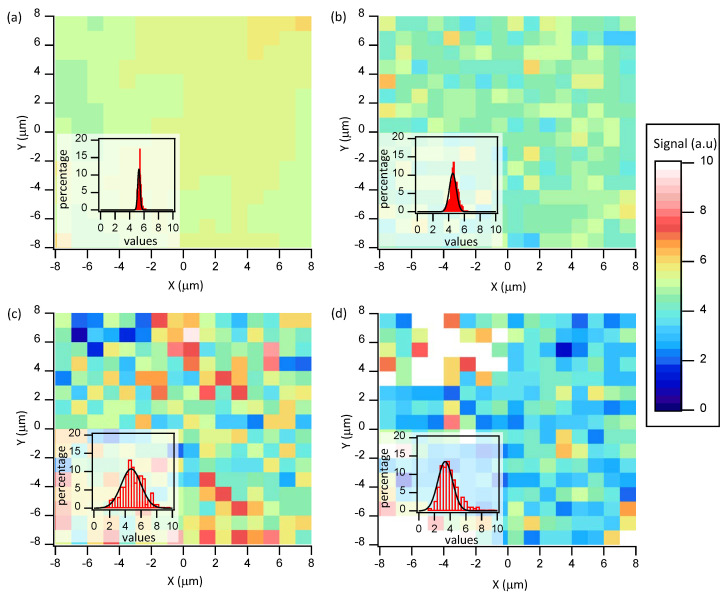
SERS scan of the 1588 cm${}^{-1}$ peak for 4-MBA adsorbed on different gold NP monolayers prepared at the optimal lateral pressure and an electroless plating time of 8 min. Data correspond to gold nanostars (**a**), 10 nm gold NP (**b**), 5 nm gold NP (**c**), and 30 nm gold NP (**d**). The distribution of intensities is plotted as an inset in each case, and a Gaussian is fitted to each distribution as a guide for the eye.

To assess the quality of the fabricated NP substrates, we have measured the homogeneity of their SERS performance across the sensing surface by mapping the SERS intensity for the 1588 cm${}^{-1}$ peak of 4MBA in a region of size 15 μm × 15 μm, at 1 μm steps. Indeed, the 4MBA form a monolayer on the nanoparticles surface and its SERS signal gives a reliable indication of the density of hotspots presents and their overall contribution to the measurement. For each 1μm${}^{2}$ area, we integrated the SERS signal from 1530 to 1625 cm${}^{-1}$ (Figure 5). We report this analysis for monolayers of nanostars and NP of 5 nm, 10 nm, and 30 nm, prepared at the optimal performance conditions (see above). In all cases, we characterize the noisy pattern in the distribution of SERS intensities by computing their statistical distribution after we normalize the range of signal intensities to compare all substrates. We find that the signal variance of the nanostar substrate is the lowest of all tested samples, with a dispersion of around 3% over the explored area (Figure 5a). In the case of spherical NP, the lowest variance is obtained for 10nm NP at around 8% (Figure 5b), increasing to more than 20% for smaller (Figure 5c) or larger NP (Figure 5d). It is often speculated that this signal dispersion is due to inhomogeneities in the electroless plating process or to the presence of traces of organic impurities other than 4-MBA. However, here we have performed a systematic study where all protocols are identical except for the size or shape of the employed NP, finding a trend that merits some explanation.

The presence of chemical impurities can be ruled out as a main cause for the signal dispersion, given the uniformity of the used protocols, so we should focus on the effect of electroless plating. For small NP, where the lateral packing at optimal performance is the highest (see Figure 2a), electroless plating quickly leads to merging of neighboring NP. This random process should contribute to the formation of a wide range of effective hotspot sizes, thus leading to an increase in the SERS variance. On the other hand, for large NP, the optimal surface pressure corresponds to a sparse lateral packing (see Figure 2b), which implies an intrinsic signal variance that appears in the mapped SERS spectra. Interestingly, the variance seems to be minimized for 10nm NP, which we have seen to be at near optimal performance for spherical NP. Therefore, we have found a link between optimal performance of SERS substrates and minimal signal variance across the surface, an idea that is further validated by the observations with nanostars. These substrates have both the highest performance and lowest signal variance of all tested NP. Although a clear understanding of this phenomenon is not readily available, this result deserve further analysis in a future work. Moreover, it indicates an additional advantage of the LB technique with respect to simpler self-assembly protocols. With LB, the regularity in lateral packing can be optimized though NP functionalization, aqueous subphase modification, and a careful tailoring of the compression/expansion protocol.

**Figure 6:**
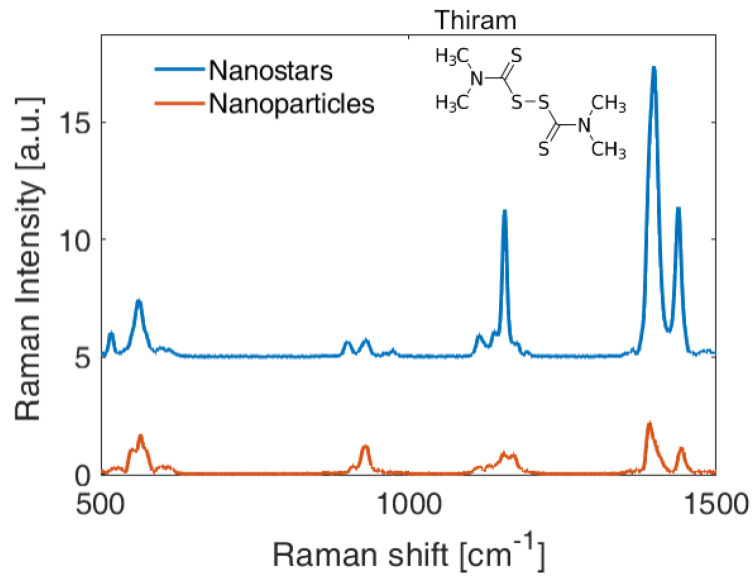
SERS signal and detection of 1 μM of Thiram in water (240 ppb) for nanostars and nanospheres-based substrates realized with the optimal pressures of respectively 13 mNm${}^{-1}$ and 10 mNm${}^{-1}.$

After finding the optimal conditions for the best SERS performance of both types of substrates and a test analyte, we have fabricated new substrates following the described protocol and tested the detection of 1 a μM solution of Thiram corresponding to 240 ppb. We chose Thiram since it is an extremely widespread fungicide and is one of the water contaminants that poses a health risk, particularly in developing countries. In Figure 6, we report the SERS signal of the Thiram solution obtained with the nanospheres (10 nm at 13 mN m${}^{-1}$) and nanostars (50 nm at 10 mN m${}^{-1}$). This comparison confirms a stronger signal for the nanostars-based substrates showing in both cases a good signal-to-noise ratio. The homogeneity in the SERS performance of the obtained substrates and their ability to detect very low concentration of water contaminants constitutes a very promising feature to move towards efficient and optimized SERS chemical sensors.

## 4. Summary and Conclusions

In this article, we have reported a systematic study of the performance of SERS substrates prepared through the tailored self-assembly of gold NP by means of the Langmuir–Blodgett technique. We have extended previous studies where we explored the effect of lateral NP packing and further in-situ hotspot growth through gold electroless plating to include here the effect of the size of the employed NP and the effect of using star-shaped NPs. For spherical NP, we estimate that optimal performance would be achieved when NPs with diameter slightly above 10 nm were used. The SERS performance is a balance between the high hotspot density achievable for small NPs that will easily coalesce during gold electroless plating and the sparse packing achievable for large NPs that will remain as individual hotspots during electroless plating. We also show that the performance limit reached with spherical NP can be surpassed when nanostars are used instead, indicating that, although gold electroless plating transforms spherical NP into rough hotspots, it is a better strategy to prepare the films using NP cores with controlled roughness. We have also tested the optimized substrates for the detection of a water contaminant (Thiram) and demonstrated the detection of a concentration of 240 ppb.

Moreover, by mapping the SERS signal of a test analyte on an area of the substrates, we find an intriguing correlation between surface smoothness and SERS performance. Although this result deserves further studies to be well understood, it highlights the interest of the Langmuir–Blodgett technique since it allows to carefully tune the lateral hotspot packing but also to optimize the homogeneity of their distribution thanks to the capillary and electrostatic interparticle repulsion experienced by NPs when spread at the water/air interface.
